# The molecular basis of OH-PCB estrogen receptor activation

**DOI:** 10.1016/j.jbc.2021.100353

**Published:** 2021-01-30

**Authors:** Ting Wang, Ian Cook, Thomas S. Leyh

**Affiliations:** Department of Microbiology and Immunology, Albert Einstein College of Medicine, Bronx, New York, USA

**Keywords:** polychlorinated biphenyl, hydroxylated PCB, sulfotransferase 1E1, inhibitor design, spin-label NMR structure, molecular dynamics, estrogen-receptor activation, 1-HP, 1-hydroxypyrene, DTNB, 5,5′-Dithiobis(2-nitrobenzoic acid), E2, 17-beta-estradiol, ER, estrogen receptor, OH-PCB1, 4ʹ-OH-2,6-dichlorobiphenol, OH-PCB2, 4-OH-3,3',4',5-tetrachlorobiphenol, PAP, 3′- phosphoadenosine 5′-phosphate, PAPS, 3′-phosphoadenosine 5′-phosphosulfate, PCB, polychlorinated biphenyls, pNpp, para-nitrophenylphosphate, SULT, sulfotransferase, TCE, 2,2,2-trichloroethanol

## Abstract

Polychlorinated bisphenols (PCBs) continue to contaminate food chains globally where they concentrate in tissues and disrupt the endocrine systems of species throughout the ecosphere. Hydroxylated PCBs (OH-PCBs) are major PCB metabolites and high-affinity inhibitors of human estrogen sulfotransferase (SULT1E1), which sulfonates estrogens and thus prevents them from binding to and activating their receptors. OH-PCB inhibition of SULT1E1 is believed to contribute significantly to PCB-based endocrine disruption. Here, for the first time, the molecular basis of OH-PCB inhibition of SULT1E1 is revealed in a structure of SULT1E1 in complex with OH-PCB1 (4ʹ-OH-2,6-dichlorobiphenol) and its substrates, estradiol (E2), and PAP (3’-phosphoadenosine-5-phosphosulfate). OH-PCB1 prevents catalysis by intercalating between E2 and catalytic residues and establishes a new E2-binding site whose E2 affinity and positioning are greater than and competitive with those of the reactive-binding pocket. Such complexes have not been observed previously and offer a novel template for the design of high-affinity inhibitors. Mutating residues in direct contact with OH-PCB weaken its affinity without compromising the enzyme’s catalytic parameters. These OH-PCB resistant mutants were used in stable transfectant studies to demonstrate that OH-PCBs regulate estrogen receptors in cultured human cell lines by binding the OH-PCB binding pocket of SULT1E1.

The human cytosolic sulfotransferase (SULT) enzyme family comprises 13 broad-specificity isoforms that operate in distinct yet partially overlapping metabolic areas. SULTs catalyze regiospecific transfer of the sulfuryl moiety (-SO_3_) from PAPS (3’-phosphoadenosine-5’-phosphosufate) to the hydroxyls and amines of hundreds, perhaps thousands of endo- and xenobiotics including scores of signaling small molecules and FDA-approved drugs (1). Attachment of the sulfuryl group at a specific site in a small-molecule recodes its functions by altering its interactions with cognate-binding site(s) and can lead to enhancements in solubility and transport that determine its terminal half-life (2). Normal functioning of numerous cellular processes depends on a single, critically positioned sulfuryl-group—steroid- (3, 4, 5), peptide- (6), dopamine- (7), and thyroid- (8) receptors, the immune system (9), lymph circulation (10), homeostasis (11), pheromone reception (12), and growth factor recognition (13).

The SULT1E1 isoform plays a pivotal role in regulating the cellular activities of estrogens. The sulfuryl moiety prevents estrogens from binding to and activating their receptors (14, 15). Consequently, SULT1E1 activity is linked to physiological processes in which estrogens are engaged, which positions the enzyme as a potential therapeutic target in circumstances where enhanced estrogenic activity (by inhibiting its inactivation) is desirable (*e.g.*, metabolic syndrome (16), diabetes (17), renal failure (18), and estrogen therapeutic augmentation (19)).

Hydroxylated polychlorinated biphenyls (OH-PCBs) are the most potent known SULT1E1 inhibitors—K_i OH-PCB_ values range as low as ∼100 pM (20). OH-PCBs derive metabolically from their parent PCBs, which are highly stable, lipophilic, environmental toxins that bioaccumulate (21). PCBs have entered ecosystems and food chains on a global scale where they disrupt the endocrine signaling systems of numerous species (22, 23, 24), including humans (25, 26). OH-PCB half-lives in human serum range from 2.6 to 15 years (27), and PCBs will persist in our environment for centuries (27). Approximately 2 billion kg of PCBs were produced between early 1920 and late 1970 (28), when they were banned (29). Remediation is ongoing at 16 PCB-contamination Superfund sites in the United States (30).

To better understand the molecular basis of OH-PCB action, and with the intent to use the findings as a template for the design and synthesis of potent SULT1E1-specfic inhibitors, we determined the solution structure of SULT1E1 bound to PAP, estradiol (E2), and OH-PCB1 (Fig. 1). Structure and binding studies reveal that OH-PCB1 binds to and reshapes the SULT1E1 active site into a high-affinity E2-binding pocket in which E2 interacts directly with OH-PCB1 and cannot access catalytic residues. Finally, structurally guided SULT1E1 mutagenesis is used to demonstrate that estrogen receptor (ER) activation in cultured human cells is regulated by OH-PCB binding to SULT1E1.

**Figure 1:**
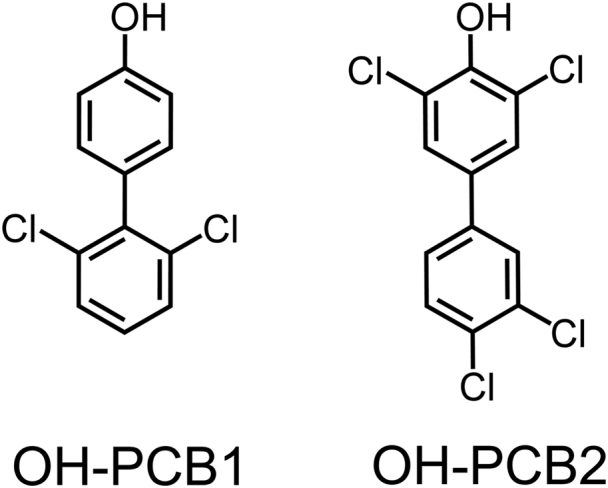
**Hydroxylated PCBs used in this study.** OH-PCB1, 4ʹ-OH-2,6-dichlorobiphenol, and OH-PCB2, 4-OH-3,3',4',5-tetrachlorobiphenol.

## Results and discussion

### OH-PCB selection

The OH-PCBs used in our studies are shown in Figure 1. OH-PCB1 was selected because its exchange rate is well suited to the NMR line-broadening methods used in the structural studies described below. Notably, OH-PCB1 is present at high levels in the serum of individuals living in Japan (21). OH-PCB2 was chosen because it is among the highest affinity SULT1E1 inhibitors known (K_i_ ∼ 400 PM (20)) and it derives from one of the most prevalent PCB contaminants in the United States (31, 32), PCB77 (33).

### Structure determination strategy

The effects of an unpaired spin-label electron on the solution NMR spectrum of a bound ligand in rapid exchange (34, 35, 36) with bulk solvent are well established. Electron/nuclear spin–spin interactions broaden NMR linewidths in a distance-dependent fashion, which is given by the Solomon–Bloembergen equation (37, 38). When distances are measured from three well-spaced spin labels located at defined positions on the protein scaffold, each ligand proton can be triangulated and thus positioned on the scaffold. The structure of the enzyme⋅ligand complex is then refined using NMR-distance-constrained molecular dynamics (MD) docking and confirmed *via* mutagenesis.

### Spin label attachment sites

Spin label attachment sites were selected from SULT1E1 backbone regions predicted to be stable by MD evaluation of the fully equilibrated E⋅PAPS⋅E2 scaffold. Surface residues that are solvent exposed and well isolated from the catalytic machinery were selected from the stable regions using the criterion that they be separated to maximize the spin-label paramagnetic field coverage of the protein surface. Finally, all insertion points satisfied the criteria that their C_α_ RMSF be ≤1.0 Å and that the RMSF of the modeled spin-label nitroxyl-oxygen be ∼6.0 Å, suggesting unfettered motion.

Spin labels were attached to the SULT1E1 scaffold at Cys residues that were site-specifically incorporated *via* PCR-based mutagenesis. Prior to creating spin-label attachment constructs, reactive Cys was removed from the native scaffold. Only one of the four native SULT1E1 Cys (*i.e.*, C69) reacts with DTNB (39), and mutagenic conversion of C69 to ser produced a stable, fully active non-DTNB-reactive scaffold. Five individual cys-attachment constructs were prepared from the C69S mutant (G16C, K25C, N150C, Q163C, and N233C). Mutants were labeled (see, Experimental procedures) and the initial rate parameters (k_cat_, K_m_ and K_i_) of each spin-labeled mutant were determined (see, Experimental procedures, *Initial-Rate studies*) to evaluate whether the catalytic integrity of the constructs had been compromised by the insertions and/or spin label attachments. The results, compiled in Table 1, reveal that the labeled mutant parameters are nearly identical to those of wild-type (WT) SULT1E1.

**Table 1:** Initial-rate parameters for WT and spin-labeled SULT1E1

Enzyme	k_cat_ (min^−1^)	K_m_ (nM)	K_i OH-PCB1_ (nM)
WT	49 (1.5)^a^	21 (1.8)	60 (6.8)
16^b^	55 (2.6)	25 (2.2)	63 (5.1)
150	51 (2.1)	23 (2.2)	65 (6.2)
233	50 (2.0)	21 (2.0)	58 (5.2)

a Values in parentheses indicate one standard deviation.

b Cys residue at which spin label is attached.

**Figure 2:**
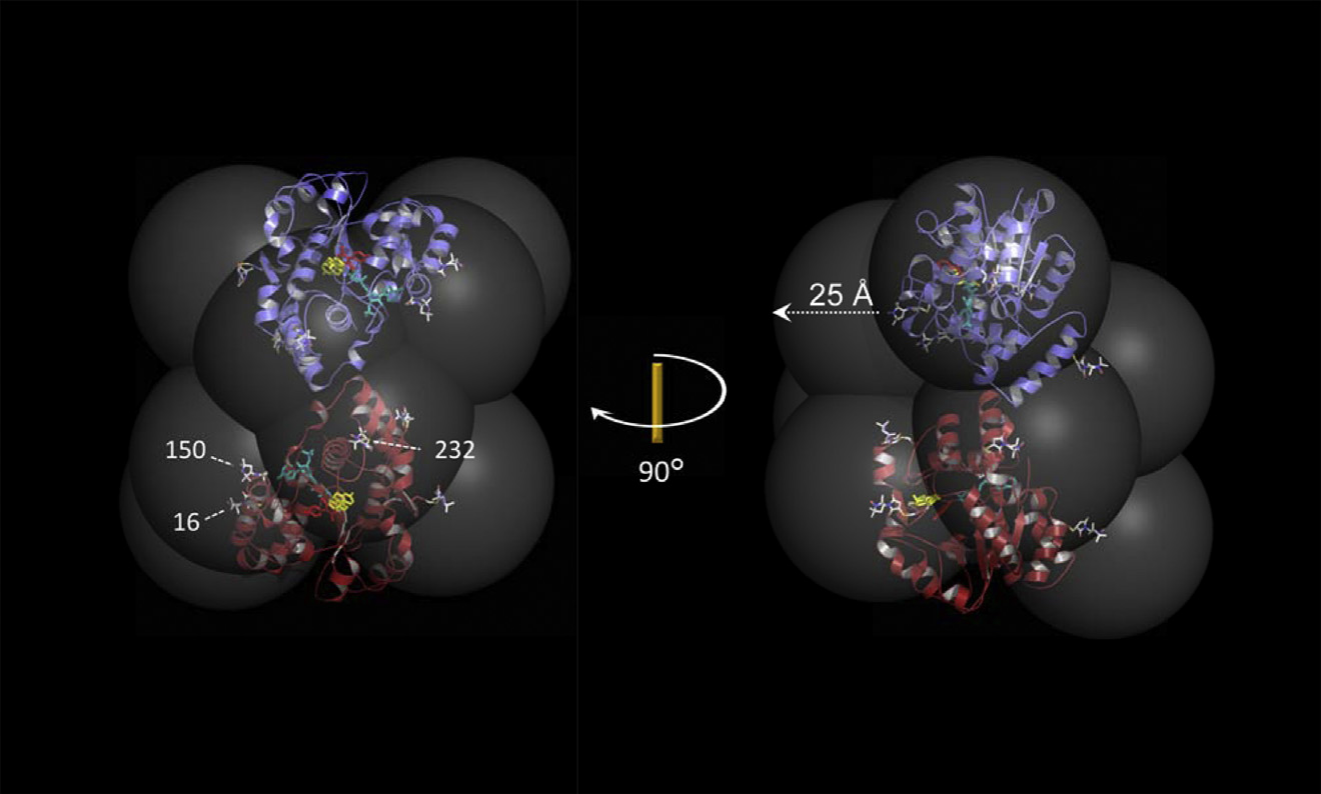
**The spin-labeled SULT1E1 constructs.** SULT1E1 subunits are shown in *blue* and *red*, and ligands, PAP, E2, and OH-PCB1, are colored *teal*, *yellow*, and *red*, respectively. *Spin labels* (*white*) are shown attached at five positions chosen to completely “coat” the dimer in a paramagnetic field of sufficient strength to detect its effects on the solution NMR spectrum of ligands without compromising the catalytic integrity of the enzyme. The experimental constructs incorporate one spin label per subunit. The three spin labels selected for structural studies are labeled according to their attachment-site sequence position. Semitransparent spheres are centered on spin label nitroxyl-oxygen atoms and their radii are set at 25 Å—the approximate maximum distance over which ligand/spin label interactions can be detected. The indicated 90° rotation transforms the left- into the right-hand structure.

The spin labels used in the current study are presented in Figure 2 (white carbon atoms). Interaction between a bound-ligand proton and an unpaired electron can be detected when the interspin distance is ≤ ∼ 25 Å. The large semitransparent spheres seen in Figure 1 are centered on the spin-label nitroxyl-moiety oxygen atom and their radii (25 Å) correspond to the approximate, maximum detectable interspin distance. As is evident, the distribution “coats” nearly the entire surface of the protein with a paramagnetic field of sufficient strength to broaden ligand ^1^H-NMR peaks, thus allowing distances to be determined, regardless of where the ligand binds. Figure 2 shows spin labels at the five attachment sites used in the initial screen. The three spin labels used in the distance studies are labeled according to their residue positions.

### NMR distance measurements

The distance-dependent effects of protein-coupled spin labels on NMR linewidths of ligands in solution are well understood (40, 41, 42). The distance (*r*) between the unpaired electron and nucleus is given by the following equation (37, 38):

(1) $$r={\left\{R_{2}/\left({\left(\frac{\mu_{o}}{60\;\pi }\right)}^{2}\gamma^{2}{\text{g}}^{2}\mu_{B}^{2}\;S\left(S+1\right)\left\{4\tau_{c}+\frac{3\tau_{c}}{1+{\left(\omega \;\tau_{c}\right)}^{2}}\right\}\right)\right\}}^{-6}$$

where *R*_*2*_ is the transverse relaxation rate of the nuclear spin, *μ*_*o*_ is the permeability of a vacuum, *γ* is the proton gyromagnetic ratio, g is the electron g-factor, *μ*_*B*_ is the magnetic moment of the free electron, *S* is the electron spin quantum number, *τ*_*c*_ is the rotational correlation time of the protein, and ω is the Larmor frequency of the proton.

**Figure 3:**
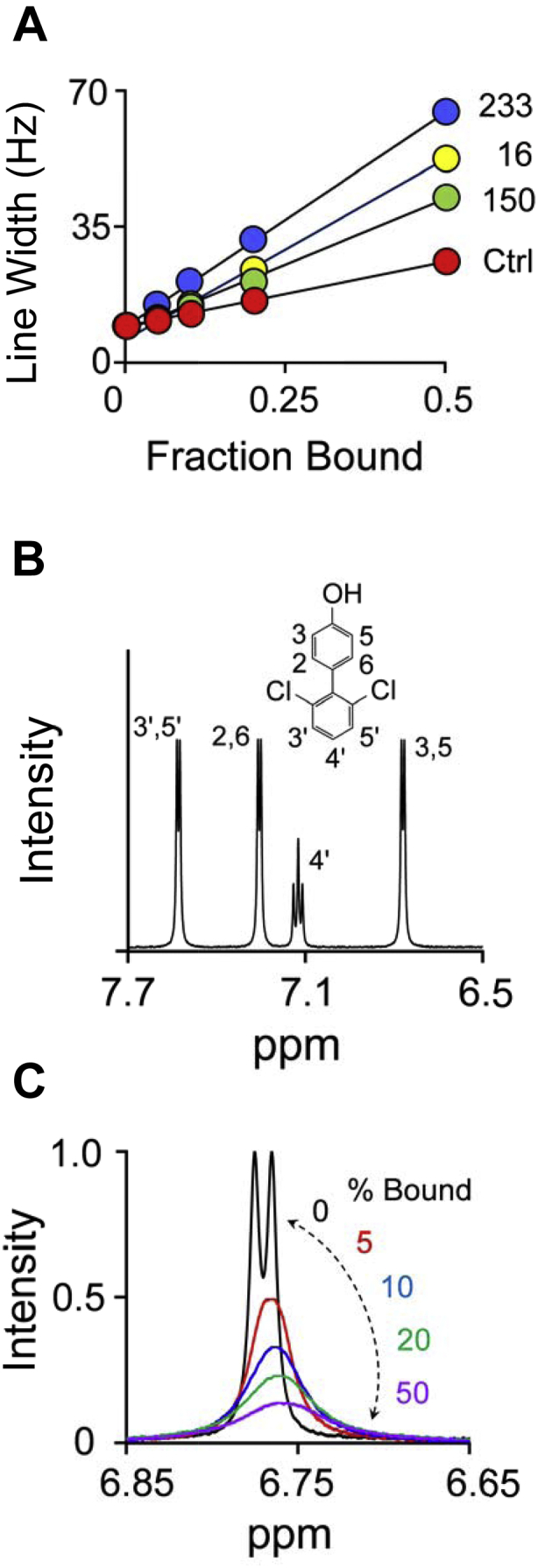
**OH-PCB1 NMR Measurements.** Panel *A*, Line-Width *versus* Fraction-OH-PCB1-Bound plots. The effects of dia- and paramagnetic SULT1E1 constructs on the linewidth of the OH-PCB1 H3 H5-proton peak are plotted *versus* the fraction of OH-PCB1 bound to the enzyme. Conditions: OH-PCB1 (50 μM), dia- and paramagnetic SULT1E1 constructs (2.5–25 μM, monomer), E2 (4.0 μM + SULT1E1 monomer concentration), PAP (300 μM, 100 × K_m_), KPO_4_ (50 mM), pD 7.4, 25 °C ± 1 deg. C. Line numbers correspond to spin-label attachment sites. The diamagnetic label in the control construct (Ctrl) is attached at position 233. Each point is the average of three independent determinations and the errors are smaller than the dot diameters. Panel *B*, OH-PCB1 structure and 600 MHz ^1^H-NMR spectrum. Conditions: OH-PCB1 (50 μM), KPO_4_ (50 mM), pD 7.4, 25 °C ± 1 deg. C. Peaks were assigned using ^1^H-^13^C HSQC and HMBC (see, Experimental procedures). Panel *C*, spin label effects on OH-PCB1 H3 H5 peak width. The OH-PCB1 H3 H5 peak is shown as a function of the percent of OH-PCB1 bound to spin-labeled C233-SULT1E1. Conditions: OH-PCB1 (50 μM), spin-labeled C233-SULT1E1 (0, 2.5, 5.0, 10, 25 μM monomer—*black*, *red*, *blue*, *green*, and *purple*, respectively), E2 [4.0 μM (1000 × K_d_ for the E⋅PAP⋅OH-PCB1 complex) + monomer concentration], PAP (300 μM, 100 × K_m_), KPO_4_ (50 mM), pD 7.4, 25 °C ± 1 deg. C. The lowest free concentration of OH-PCB1 (25 μM) is 420-fold higher than its K_i_.

Transverse relaxation rates are calculated from NMR linewidths, which, for protein-bound ligands, are typically too broad to determine accurately. If the ligand exchanges between the protein and solution at a rate comparable with or greater than the difference in Larmor frequency between the bound and free species, observed R_2_ values (R_2__*obs*_) for the bound species can be obtained from the slopes of *Solution-Phase-Linewidth versus Fraction-Ligand-Bound* plots (*i.e.*, *LW*-*versus*-*FB* plots) (43, 44). The *LW*-*versus*-*FB* plot for the H3 H5 NMR peak of OH-PCB1 is presented in Figure 3*A*. The OH-PBC1, PAP, and E2 concentrations used are reported in the Figure 3 legend. The full OH-PCB1 ^1^H-NMR spectrum and the line-broadening effect of spin label 233 on the H3 H5 peak width as a function of percent of bound ligand are given in Figure 3, *B* and *C*, respectively. R_2 *obs*_ values contain contributions from relaxation caused by the unpaired electron (the paramagnetic contribution) and the protein (the diamagnetic contribution). Interspin distance calculations (Equation 1) depend only on the paramagnetic contribution (R_2_), which is obtained by subtracting the diamagnetic contribution from R_2__*obs*_. The diamagnetic contribution is given by the slopes of *LW*-*versus*-*FB* plots constructed using control constructs in which the spin-label PROXYL-moiety (2,2,5,5-tetramethyl-1-pyrroli- dinyloxy) is replaced by the cyclohexyl-group (42) (see, Experimental procedures).

OH-PCB1 exhibits four ^1^H-NMR resonances, three of which correspond to pairs of chemically indistinguishable protons (H3/H5, H2’/H6’, and H3’/H5’). The 12 interspin distances needed to triangulate the protons associated with the four resonances were obtained from *LW-versus-FB* plots (see, Fig. 3*A* and Fig. S1) and are given in Table 2.

**Table 2:** Proton to spin-label distances (Å)

Proton	Spin label attachment residue		
	16	150	233
4ʹ	19 (3)^a^	22 (4)	24 (3)
3ʹ 5ʹ	20 (3)	25 (4)	22 (3)
2 6	22 (3)	24 (3)	16 (3)
3 5	28 (4)	25 (4)	14 (2)

a Values in parentheses indicate 95% confidence interval.

### Refining the structure

**Figure 4:**
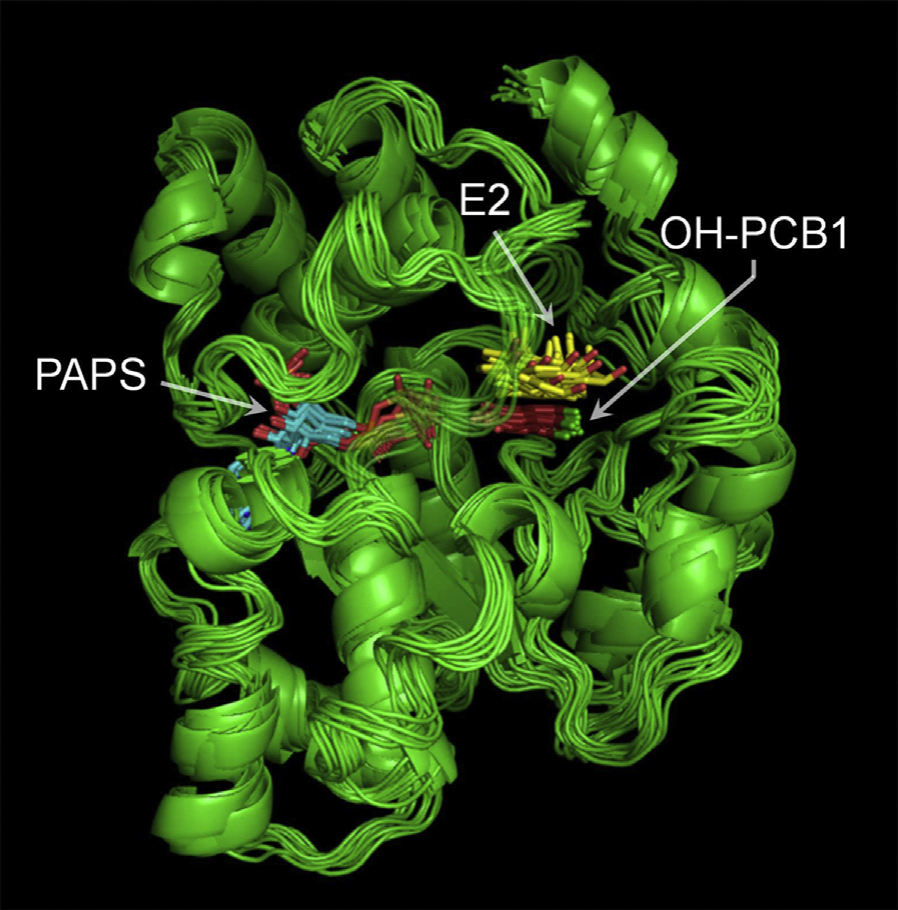
**NMR-distance-constrained docking of OH-PCB1 to SULT1E1 ⋅PAPS⋅E2.** Docking and cluster analysis were performed with GROMACS (see, Experimental procedures). OH-PCB1 docking was repeated ten times. *g-cluster* analysis of the ten structures detected a single cluster (≤2.0 Å RMSD). All ten structures are superposed in the figure and are available for download at ModelArchive (Accession No. ma-xnec7).

NMR triangulation distances were used in conjunction with MD docking simulations (45, 46) to obtain the structure of the E⋅PAPS⋅E2⋅OH-PCB1 complex. During the simulations, each proton is constrained by a restoring force (50 kJ mol^−1^ Å^−1^) to move within an ellipsoid whose centerpoint is given by the intersection of the three NMR distance vectors that position it and whose principal axes lengths correspond to the standard errors (±1 σ) of the distance measurements. The restoring force (applied using *distance_restraints* in GROMACS) drives a proton toward the center of its ellipsoid if it lies outside of the ellipsoid surface (45, 46). Given that OH-PCB1 contains three proton pairs that are indistinguishable by ^1^H-NMR, distance constraints were applied not to each proton in a pair, but to the midpoint of the chord that connects the pair. As is appropriate for NMR distance measurements (37, 38, 47), *distance _restraints* was parameterized to use time-averaged, (1/r^6^)-weighted distance restrains, which were applied simultaneously to the 12 distances that constrain the four OH-PCB1 positions. Docking simulations were run long enough to allow structures to achieve equilibrium. The results of ten simulations were analyzed using *g_cluster* in GROMACS. Only a single structural cluster (≤2.0 Å RMSD) was detected. The resulting structures were virtually identical and are overlain in Figure 4. The structures can be downloaded at ModelArchive (48) (accession # ma-xnec7). No significant structural changes were detected over 10 ns once the distance constraints were removed.

### The structure

**Figure 5:**
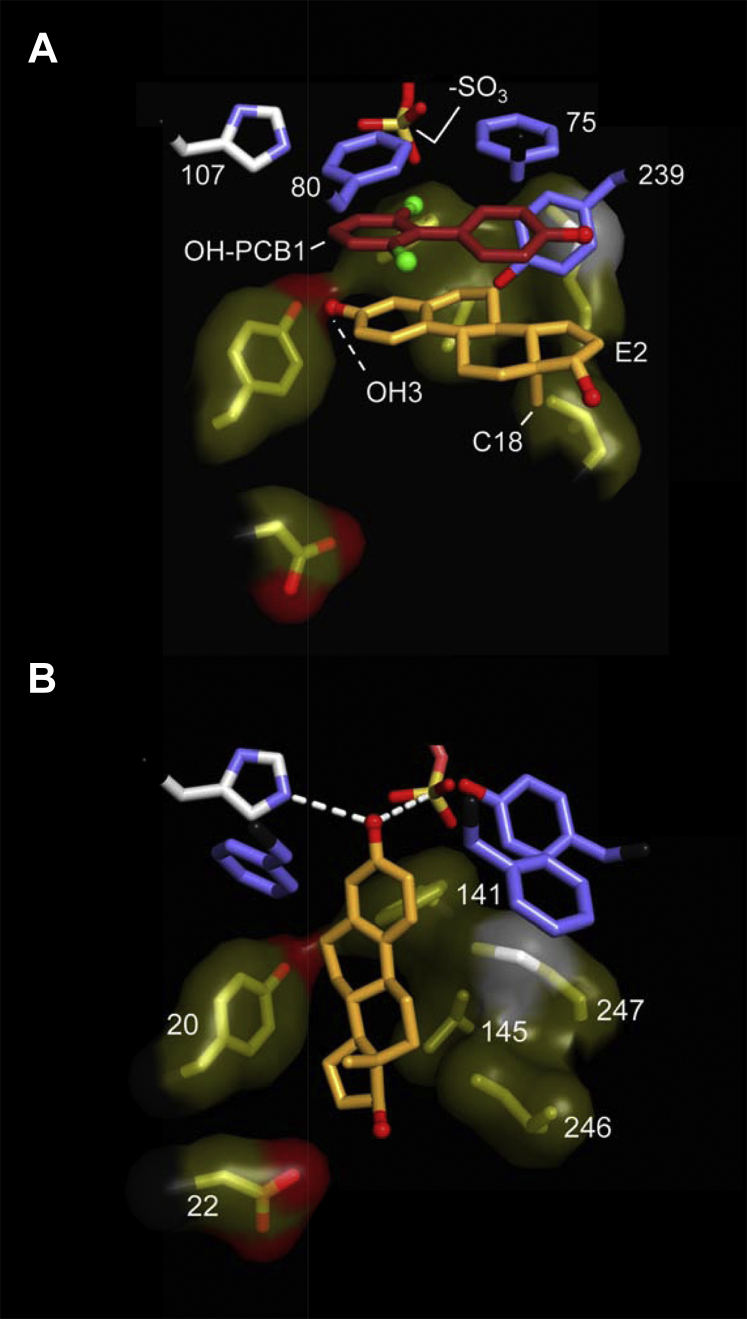
**SULT1E1 active-site structure (+/−) OH-PCB1.** Panel *A*, the E⋅PAPS⋅E2⋅OH-PCB1 complex. OH-PCB1 and E2 carbon atoms are shown in *orange* and *brick*. *Blue residues* are in direct contact with OH-PCB1. The PAPS sulfuryl (-SO_3_) moiety is labeled, as are the E2 nucleophilic hydroxyl (-OH3) and C18 methyl group. His107 is presented in identical orientations in Panels *A* and *B*. Panel *B*, the E⋅PAPS⋅E2 complex. The olive residues are in direct contact with E2, the *blue residues* are not. Residues in Panels *A* and *B* are labeled according to their sequence positions.

The structure of OH-PCB1 bound to its binding site in the E⋅PAPS⋅E2⋅OH-PCB1 complex is presented in Figure 5*A*. OH-PCB1 is situated in a highly hydrophobic pocket and is in direct contact with the three residues highlighted in blue (P75, P80, and Y239). The OH-PCB1 benzyl rings are sandwiched on one side by the rings of P75 and P80 and on the other by those of E2. The ring plane of Y239 contacts edges of both OH-PCB1 and E2. Remarkably, the binding of OH-PCB1 establishes a new E2-binding site in which it prevents catalysis by intercalating between E2 and the catalytic machinery. The structure reveals how an inhibitor (OH-PCB1) that sterically prevents a substrate (E2) from binding its active-site pocket can be transformed from a competitor to an allostere whose effects cannot be diminished by increasing substrate concentrations.

In moving from Panel A to B, one sees the effect of withdrawing OH-PCB1—the olive residues cluster into direct-contact positions with E2, which rotates longitudinally about the axis connecting its 3- and 17-OH moieties (causing its C18-methyl group to face the viewer) and vertically, which enhances 3-OH reactivity *via* H-bonding with His107 (49) and reactive proximity (∼3.4 Å) to the sulfuryl-moiety. The structure seen in Panel B was MD-generated as previously described (50) and is virtually identical to the E⋅PAP⋅E2 structure from which it was derived (49).

### Validating the structure

**Figure 6:**
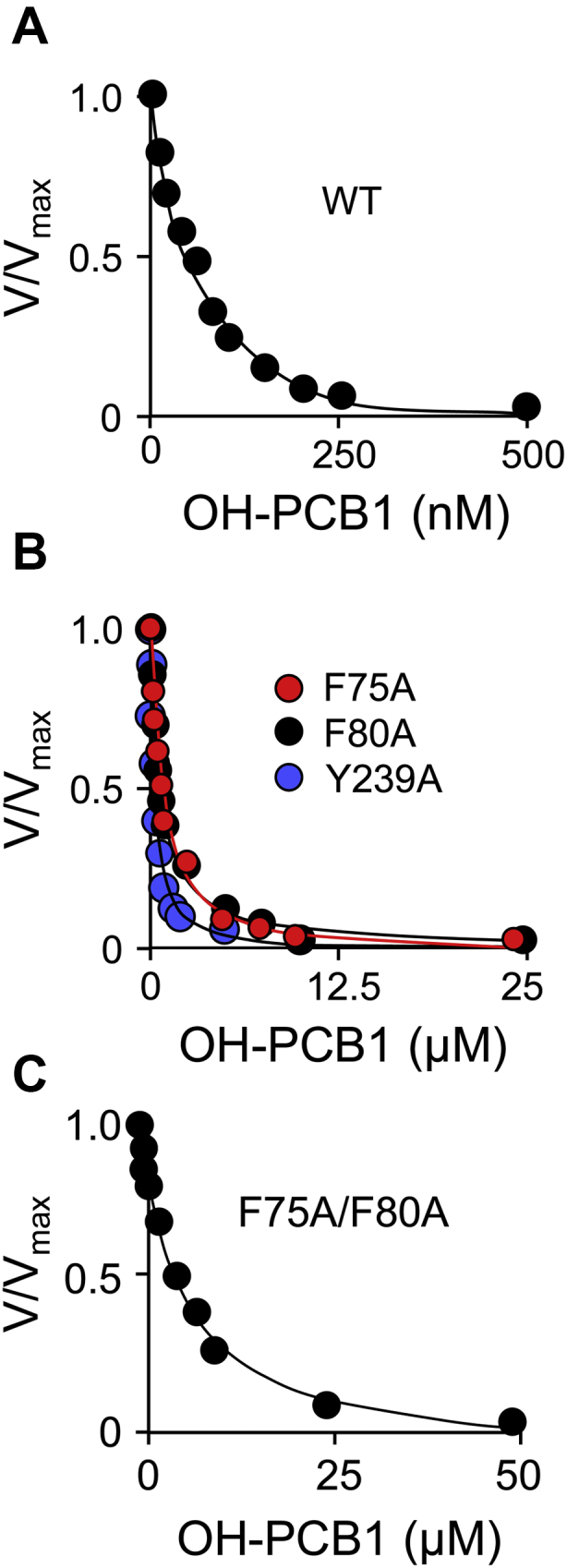
**OH-PCB1 inhibition of WT and mutant SULT1E1.** Panel *A*, inhibition of WT SULT1E1. Panel *B*, inhibition of single-mutant SULT1E1 constructs. Panel *C*, inhibition of the double-mutant SULT1E1 construct. Panels *A*–*C*, conditions: SULT1E1 (2.0 nM, active sites), OH-PCB1 (concentrations as indicated), 1-HP (2.0 μM, 100 × K_m_), PAPS (0.30 mM, 100 × K_m_), KPO_4_ (50 mM), pH 7.5, 25 °C ± 2 deg. C. Reaction progress was monitored *via* the fluorescence change associated with 1-HP sulfonation (λ_ex_ = 325 nm, λ_em_ = 370 nm). Rate measurements were taken during conversion to product of ≤5% of the concentration limiting substrate consumed at the reaction endpoint. Rates were normalized to the rate in the absence of inhibitor (*i.e.*, V/V_max_). Each point is the average of three independent measurements and the associated standard deviations are smaller than the dot diameters. Averaged data were least-squares fit to a noncompetitive inhibition model (see, Experimental procedures) and the lines passing through the data represent the behavior predicted by the best-fit constants reported in Table 3.

In the absence of inhibitor, the olive residues are in direct contact with E2, and the blue residues lie slightly beyond E2-contact distance, suggesting that their contribution to substrate binding and catalysis might be slight. If the OH-PCB1-binding and catalytic functions of the site are separable, the effects of mutating the OH-PCB1 binding site on the OH-PCB1 inhibition constant (K_i OH-PCB1_) can be used to validate the structure. To assess whether mutagenizing OH-PCB1 binding-site residues influences the catalytic functions of SULT1E1, the effects of such mutations on the initial-rate parameters (K_m_ and k_cat_) of 1-hydroxypyrene (1-HP, a fluorescent E2-like substrate (51, 52)) were determined (see, Experimental procedures). OH-PCB1 direct-contact residues (F75, F80, and Y239) were mutated to alanine (43, 44) individually and in combination (F75/F80), and the results of the initial-rate studies are given in Figure 6, *A*–*C* and Table 3. As is evident, the mutations have no measurable effect on catalytic function (*i.e.*, K_m 1-HP_ and k_cat_). In contrast, the mutations had pronounced effects on K_i OH-PCB1_. Mutating Y239, which contacts an edge of OH-PCB1, causes a 5.7-fold decrease in affinity, while mutating residues that sandwich the inhibitor (F75 and F80) result in larger decreases (11- and 12-fold, respectively). The effect of the F75/F80 double mutation, 113-fold, is within error equal to the product of the individual mutations, suggesting that these residues operate largely independently. These findings fully support the structure seen in Figure 5*A*, and confirm that the OH-PCB1-binding and catalytic functions of SULT1E1 are indeed separable.

**Table 3:** Initial-rate effects of mutating OH-PCB1 direct-contact residues

Enzyme	K_m 1-HP_ (nM)	k_cat_ (min^−1^)	K_i OH-PCB1_ (nM)^a^	Fold Effect^b^
WT	25 (2.2)^c^	38 (2.2)	0.060 (0.007)	1.0
F75A	27 (1.7)	36 (2.1)	0.63 (0.04)	11
F80A	23 (1.8)	35 (1.7)	0.73 (0.07)	12
Y239A	25 (2.3)	38 (2.2)	0.34 (0.03)	5.7
F80A/F75A	28 (2.2)	35 (2.3)	6.8 (0.9)	113

a Values were obtained by least-squares fitting using a noncompetitive inhibition model (see, Experimental procedures).

b Fold Effect = K_i OH-PCB1_/K_i OH-PCB1 WT_.

c Values in parentheses indicate one standard deviation.

### The energetics of OH-PCB/reactant interactions

**Figure 7:**
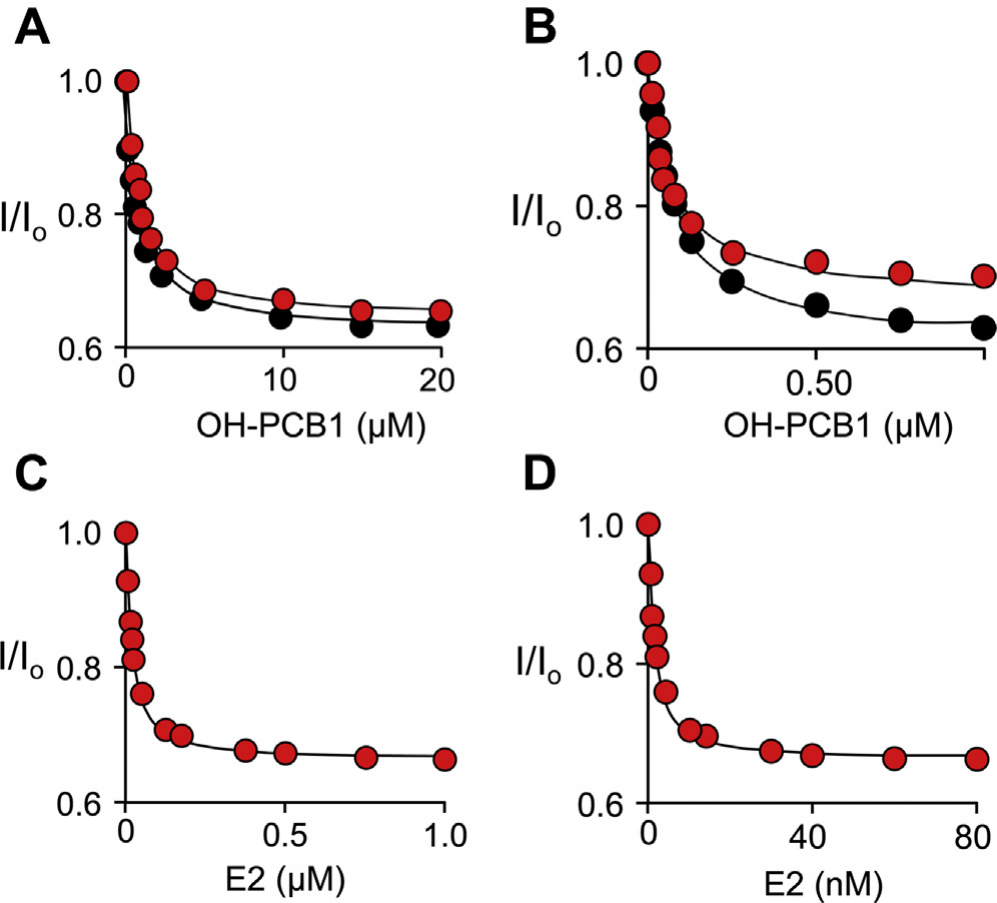
**OH-PCB1 and E2 binding to SULT1E1 complexes.** Panel *A*, OH-PCB1 binding to E and E⋅PAP. Conditions: SULT1E1 (50 nM, active sites), OH-PCB1 (0.25–20 μM), PAP (0, *black dots*, or 300 μM (100 × K_d_) *red dots*), KPO_4_ (50 mM), pH 7.4, 25 °C ± 2 deg. C. Panel *B*, OH-PCB1 binding to E⋅E2 and E⋅E2⋅PAP. Conditions: SULT1E1 (15 nM, active sites), OH-PCB1 (12.5–1000 nM), E2 (4.0 μM), PAP (0, *black dots*, or 300 μM (100 × K_d_) *red dots*), KPO_4_ (50 mM), pH 7.4, 25 °C ± 2 deg. C. Panel *C*, E2 binding to E⋅PAP. Conditions: SULT1E1 (10 nM, active sites), E2 (1.0–1000 nM), PAP (300 μM, 100 × K_d_), KPO_4_ (50 mM), pH 7.4, 25 °C ± 2 deg. C. Panel *D*, E2 binding to E⋅PAP⋅OH-PCB1*.* Conditions: SULT1E1 (10 nM, active sites), E2 (0.50–80 nM, 0.10–40 × K_d_), OH-PCB1 (10 μM, 150 × K_d_), PAP (300 μM, 100 × K_d_), KPO_4_ (50 mM), pH 7.4, 25 °C ± 2 deg. C. Panels *A*–*D*, binding was monitored *via* ligand induced changes in SULT1E1 intrinsic fluorescence (λ_ex_ = 290 nm, λ_em_ = 345 nm). Fluorescence intensity is given relative to that in the absence of titrant (I/I_0_). Titrations were performed in triplicate and associated standard deviations are smaller than the dot diameters. Lines passing through the data represent least-squares fits of the averaged data to the following binding model: I_o_ − ΔI∙(([L]+E_tot_ + K_d_) − [([L]+E_tot_ + K_d_)^2^ − (4∙[L]∙E_tot_)]^1/2^)/2∙E_tot_, where I_o_ and ΔI represent fluorescent intensity at zero and infinite [L].

To better understand the inhibition mechanism, interactions between OH-PCBs and reactants were probed using equilibrium binding studies. OH-PCB-binding titrations were performed at saturating reactant concentrations and monitored *via* ligand-binding induced changes in SULT1E1 intrinsic fluorescence (see, Experimental procedures). OH-PCB1 titrations are presented in Figure 7, *A* and *B*, and the associated dissociation constants are given in Table 4. Consistent with the structure, OH-PCB1 and PAP do not detectibly interact—OH-PCB1 affinities for E and E⋅PAP are essentially identical (750 ± 27 and 780 ± 40 nM, respectively). In contrast, and as is supported by their contact in the structure, OH-PCB1 and E2 influence one another’s affinities—OH-PCB1 binds ∼12-fold more tightly to E⋅E2 than to E (62 ± 2 *versus* 750 ± 27 nM). Notably, PAP binding does not alter the energetics of OH-PCB1/E2 interactions—OH-PCB1 affinities for E⋅E2 and E⋅E2⋅PAP are experimentally indistinguishable (62 ± 2 and 66 ± 3 nM).

**Table 4:** Ligand dissociation constants

Enzyme species^a^	Ligand		
	OH-PCB1	OH-PCB2	E2
	K_d_ (nM)		
E	750 (27)^b^	7.2 (0.6)^e^	-
E⋅PAP	780 (40)	-	13 (1)^c^
E⋅E2	66 (3)	-	-
E⋅PAP⋅E2	62 (2)^d^	0.40 (0.18)^e^	-
E⋅PAP⋅OH-PCB1	-	-	0.98 (0.10)^c^
E⋅PAP⋅OH-PCB2	-	-	1.1 (0.1)^d^

a Species to which ligand binds.

b Values in parentheses indicate one standard deviation.

c,d,e Values derive from Figures 7^c^ and 8^d,e^.

d Values refer to the higher partner concentration (see, Main Text).

The energetics studies are consistent with the structure and reveal an OH-PCB1/E2 interaction energy that enhances the affinities of both ligands ∼12-fold. E2 affinities for E⋅PAP and E⋅PAP⋅OH-PCB1 were determined (13 ± 1 and 0.98 ± 0.10 nM), respectively (Fig. 7, *C* and *D*, and Table 4) and reveal that E2 binds more tightly as an inhibitor than as a substrate (K_m E2_ = 5 nM (4)).

**Figure 8:**
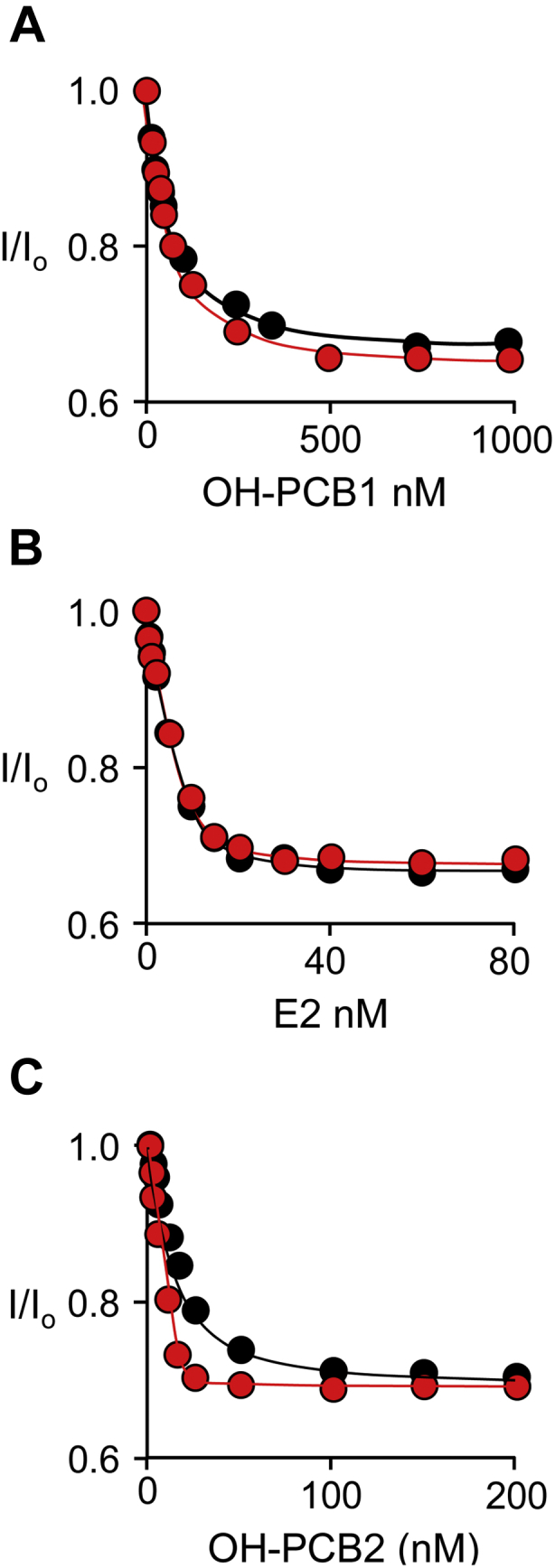
**Order-of-binding studies.** Panel *A*, OH-PCB1 Binding to E⋅PAP⋅E2. Conditions: SULT1E1 (50 nM, active sites), OH-PCB1 (10–1000 nM, 0.20–20 × K_d_), E2 (0.40 [*Black*] or 4.0 [*Red*] μM, 32 or 320 × K_d_), PAP (0.30 mM, 100 × K_m_), KPO_4_ (50 mM), pH 7.4, 25 °C ± 2 deg. C. Panel *B*, E2 Binding to E⋅PAP⋅OH-PCB2. Conditions: SULT1E1 (10 nM, active sites), E2 (0.50–80 nM, 0.10–40 × K_d_), OH-PCB2 (0.70 μM (100 × K_d_, *black dots*), or, 7.0 μM (1000 × K_d_, *red dots*)), PAP (0.30 mM, 100 × K_d_), KPO_4_ (50 mM), pH 7.4, 25 °C ± 2 deg. C. Panel *C*, OH-PCB2 binding to E and E⋅PAP⋅E2. Conditions: SULT1E1 (15 nM, active sites), OH-PCB2 (0.50–200 nM, 0.20–30 × K_d_), E2 (0 μM (*black dots*) or 4.0 μM (320 × K_d_, *red dots*)), PAP (0 mM (*black dots*) or 0.30 mM (100 × K_m_, *red dots*)), KPO_4_ (50 mM), pH 7.4, 25 °C ± 2 deg. C. Panels *A*–*C*, binding was monitored *via* ligand induced changes in SULT1E1 intrinsic fluorescence (λ_ex_ = 290 nm, λ_em_ = 345 nm). Fluorescence intensity is given relative to the intensity in the absence of titrant (I/I_0_). All titrations were performed in triplicate and the associated standard deviations are smaller than the dot diameters. Lines passing through the data represent least-squares fits of the averaged data to the following binding model: I_o_ − ΔI∙(([L]+E_tot_ + K_d_) − [([L]+E_tot_ + K_d_)^2^ − (4∙[L]∙E_tot_)]^1/2^)/2∙E_tot_, where I_o_ and ΔI represent fluorescent intensity at zero and infinite [L]. Lines passing through the data represent the behavior predicted by the best-fit constants.

To assess whether OH-PCBs can add directly to the E⋅E2 complex, and vice versa, the order of binding OH-PCB and E2 was determined. To do so, the affinity of each ligand was assessed at two saturating and tenfold different concentrations of the partner ligand. If binding is ordered, the ligand’s affinity will appear to increase tenfold as the partner draws it onto the enzyme. If instead, binding is random, the ligand affinity will not vary with the change in partner concentration. The affinity of OH-PCB1 did not vary at 32- and 320 × K_d_ concentrations of E2 (0.40 and 4.0 μM, respectively); similarly, E2 affinity did not vary at 100- and 1000 × K_d_ concentrations OH-PCB2 (0.70 and 7.0 μM, respectively)—see, Figure 8, *A*–*C* and Table 4. Hence, binding is random and the enzyme can bind either ligand and rearrange from the reactive to inhibited configuration without first dissociating its partner. Notably, OH-PCB2 was used in these studies because, unlike OH-PCB1, its solubility does not preclude the high [OH-PCB]/K_d_ ratios they require.

### PCB-based regulation of the estrogen receptor

**Figure 9:**
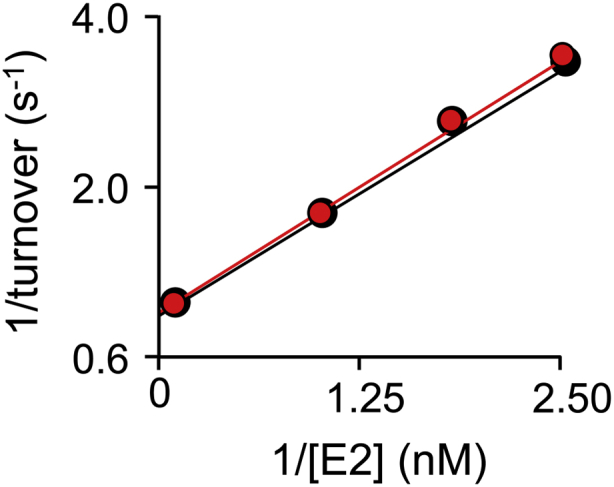
**E2 initial-rate studies.** E2 sulfation was measured *via* transfer of the sulfuryl moiety from PAPS to [^3^H]-E2. Conditions: SULT1E1 (wild-type, *black dots*; or, F75A/F80A, *red dots*, 0.10 nM active sites). [^3^H]-E2 (0.40–10 nM, 0.20–5.0 × K_m_, SA = 160 mCi μmol^−1^), PAPS (0.30 mM, 88 × K_m_), KPO_4_ (50 mM), pH 7.5, 25 °C ± 2 deg. C. Reactions were quenched by addition of KOH (100 mM final). Sulfated and nonsulfated species were separated using chloroform extraction (see, Experimental procedures). Less than 5.0% of the [^3^H]-E2 converted to product at the reaction endpoint was consumed during the rate measurements. Each point is the average of three independent measurements whose standard error is less than the dot diameters. The averaged data were least-squares fit using a (1/v^4^)-weighting. The resulting fits are given by the lines passing through the data, and the best-fit parameters are given in Table 6.

The theory that SULT1E1 inhibition plays a meaningful role in the OH-PCB-based disruption of endocrine function has not been tested directly. The ability to weaken OH-PCB affinity for SULT1E1 without influencing its catalytic properties provides an opportunity to substantiate this theory using the estrogen-response systems in human cells. Toward this end, stable transfectants that express WT or double mutant (F75/F80) SULT1E1 were constructed (see, Experimental procedures) from Ishikawa cells—an immortalized endometrial adenocarcinoma cell line (53) with undetectably low intrinsic levels of SULT1E1 expression (53). To ensure that the F75/F80 double mutation does not affect the E2 initial-rate parameters (as is the case with 1-HP), the WT and mutant E2 parameters (K_m_ and k_cat_) were determined and proved to be identical within error (see, Table 5 and Fig. 9). A (-) SULT1E1 control strain was constructed using the transfection vector without a SULT coding-region insert. Transfectants were selected for ER activation studies based on SULT1E1 activity levels in cell extracts (see, Experimental procedures). Extract activities in the WT and mutant (MT) transfectants selected for further study (4.8 and 5.0 pmol min^−1^ mg^−1^ extract, respectively) were comparable with those reported for human mammary epithelial cells (4.8 pmol min^−1^ mg^−1^ (54)) and 52-fold higher than the (-) SULT1E1 control strain extracts.

**Table 5:** E2 initial-rate parameters

Enzyme	K_m_ (nM)	k_cat_ (min^−1^)
WT	3.8 (0.2)^a^	150 (17)
F80A/F75A	4.0 (0.2)	150 (21)

a Values in parentheses indicate one standard deviation.

**Figure 10:**
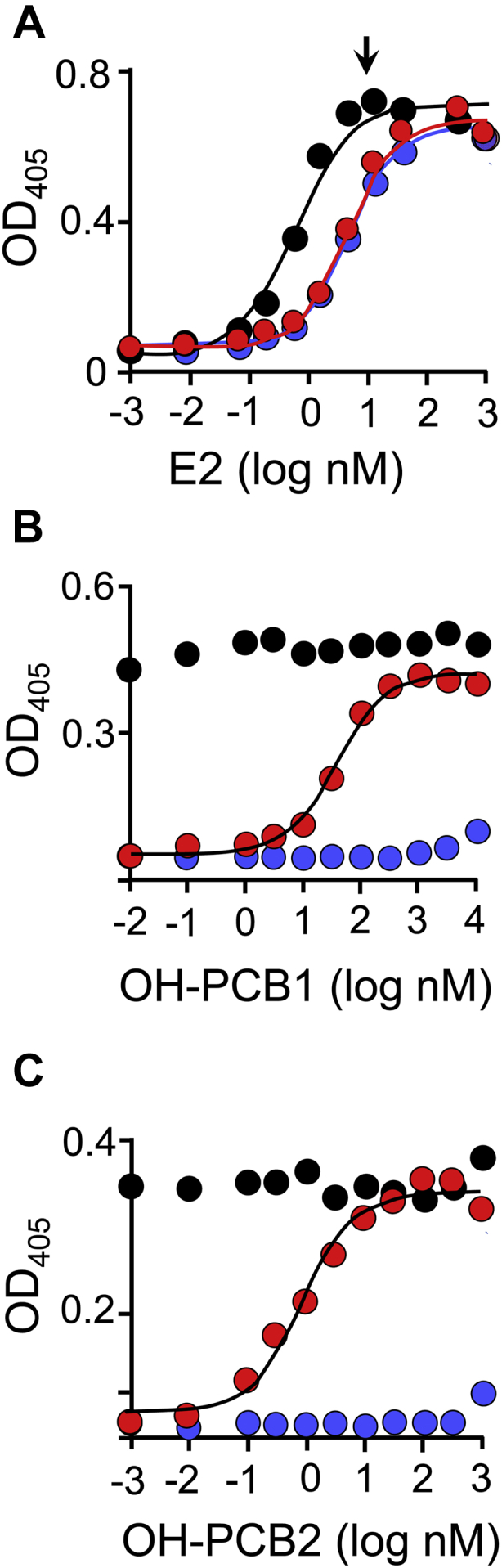
**Estrogen receptor activation studies.** Panel *A*, E2 activation. ER activation in the control (*black*), WT (*red*), and MT (*blue*) cell lines was assessed *via* alkaline phosphatase activity (see, Experimental procedures). The *arrow* pinpoints ER activation levels at the fixed E2 concentration (10 μM) used in the Panel *B* and *C* titrations. Panels *B* and *C*, OH-PCB activation studies. Conditions were identical to those in Panel *A* except that the [OH-PCB] was varied and [E2] was fixed at 10 μM. Panel *A*–*C*, titrations were performed in triplicate and averaged data were least-squares fit to the following single-site binding model: OD = OD_initial_ + ΔOD⋅([PCB]/(EC_50_ + [PCB])). *Solid lines* passing through the data are the predictions of the fits and the resulting EC_50_ values are given in Table 6.

The E2 and OH-PCB concentration dependence of transfectant ER activation was measured *via* alkaline phosphatase activity and the resulting titrations are presented in Figure 10*A*. Endogenous alkaline phosphatase levels are coordinated with ER activation and measured *via* para-nitrophenyl phosphate hydrolysis (55). Consistent with SULT1E1 inactivation of E2, the ER-activation EC_50_ of E2 is 80-fold higher in the WT (red dots) and MT (blue dots) strains, which express SULT1E1, than in the control strain (black dots), which does not (see Table 6). The arrow seen in Figure 10*A* pinpoints the ER response levels at the fixed E2 concentration (10 μM) used in the OH-PCB-titrations shown in Panels B and C. The OH-PCB1 and OH-PCB2 ER-activation patterns are similar across the three strains. OH-PCBs do not affect activation in the control strain; hence, any effects on activation in the WT and MT strains are likely linked to SULT1E1 expression. OH-PCB effects on the WT strain are pronounced—activation begins at background and increases to a maximum comparable with that associated with the control strain. OH-PCB1 and OH-PCB2 EC_50_ values (38 and 0.87 nM, respectively) are similar to their K_i_ values (60 and 0.50 nM), suggesting that the plots are reporting increases in E2 activity as SULT1E1 is inhibited. To establish that the OH-PCB effects are due to binding at the OH-PCB-binding site seen in Figure 2*A*, activation in the MT strain was tested. As is evident, activation in the MT strain remains near background throughout the entire OH-PCB concentration range with the exception of slight elevation at the highest OH-PCB concentrations, which may be due to relatively weak inhibition of the mutant and/or low-affinity OH-PCB activation of the receptor (56, 57).

**Table 6:** Estrogen receptor activation studies

Cell line	Ligand EC_50_ (nM)		
	E2	OH-PCB1^a^	OH-PCB2
Control	0.97 (0.06)^b^	NE^c^	NE
WT	80 (6)	38 (2.6)	0.87 (0.07)
F75A/F80A	76 (5)	NE	NE

a PCB EC_50_ values were determined at 10 nM E2.

b Values in parentheses indicate one standard deviation.

c NE, No Effect.

## Conclusions

A set of five SULT1E1 constructs that permit disulfide-based attachment of R-groups at catalytically innocuous sites distributed roughly uniformly across the protein’s surface has been established. Attachment of spin labels at these sites allows the entire surface of the protein to be “coated” in a paramagnetic field of sufficient strength to detect its effects on the NMR spectrum of ligands in rapid exchange, regardless of where they bind. Three spin-labeled constructs were used to determine the structure of OH-PBC1 bound to SULT1E1 in a complex with E2 and PAP. The structure, which was confirmed *via* mutagenesis, reveals that OH-PCB1 binds at the active site and forms a new E2-binding pocket in which it is sandwiched between E2 and the protein, and E2 is stabilized in a nonreactive position.

Ligand interactions and binding order were assessed in equilibrium-binding studies. Consistent with the structure, OH-PCB1 shows no net energetic interaction with PAP, but interacts significantly with E2. OH-PCB1 and E2 bind ∼12-fold more tightly in one another’s presence, leading to the conclusion that E2 binds more tightly as an inhibitor than as a substrate. Ligand binding is random, which reveals that SULT1E1 can bind either ligand and restructure from its reactive to its inactive configuration without dissociating the partner ligand.

The structure predicted that residues in direct contact with OH-PCB1 could be mutagenized to weaken OH-PCB1 binding without compromising catalysis. These predictions proved to be accurate and led to a catalytically normal, OH-PCB1-resistant double mutant that was used to demonstrate that either OH-PCB1 or OH-PCB2 can regulate estrogen receptor activation in a cultured human cell line by binding to SULT1E1. To our knowledge, this is the first direct demonstration of OH-PCB-based activation of the ER, and it supports the contention that OH-PCB inhibition of SULT1E1 contributes meaningfully to PCB-based endocrine disruption.

## Materials

The materials and sources used in this study are as follows: 5,5′-dithiobis(2-nitrobenzoic acid) (DTNB), dithiothreitol (DTT), ethylenediamine-tetraacetic acid (EDTA), L-glutathione (reduced), 1-hydroxypyrene (1-HP), Ishikawa cells, imidazole, isopropyl-thio-β-D-galactopyranoside (IPTG), lysozyme, 3-maleimido-PROXYL (2,2,5,5-tetramethyl-1-pyrrolidinyloxy), N-cyclo-hexylmaleimide, pepstatin A, potassium phosphate, and 2,2,2-trichloroethanol (TCE) were the highest grade available from Sigma. Ampicillin, fetal bovine serum (FBS), KOH, LB media, MgCl_2_, Minimum Essential Media (MEM), neomycin, pcDNA 3.1, para-nitrophenyl-phosphate (pNpp), tris(hydroxymethyl) amino-methane (Tris) base, phenylmethylsulfonyl fluoride (PMSF), radioimmunoprecipitation (RIPA) buffer, and tetramethylsilane (TMS) were purchased from Fisher Scientific. 4ʹ-OH-2,6-dichlorobiphenol and 4-OH-3,3',4',5-tetrachloro-biphenol were purchased from ChemTik. BGII and Gibson Assembly mutagenesis kits were purchased from New England Biolabs. D_2_O and D_2_-cholorform (>99%) were purchased from Cambridge Isotope Laboratories. Glutathione- and nickel-chelating resins were obtained from GE Healthcare. Lipofectamine and Opti-MEM were purchased from EMD Millipore Corporation. 17-Beta-estradiol was purchased from Steraloids. ^3^H-estradiol was purchased from PerkinElmer. Competent *E. coli* (BL21(DE3)) was purchased from Novagen. PAPS and PAP are synthesized in-house according to published protocols (58). PAPS and PAP purity, as assessed by anion-exchange HPLC, is ≥99%.

### Computer and software

MD simulations were performed on a Parallel Quantum Solutions QS32-2670C-XS8 computer. PQS Molecular Builder software was purchased from Parallel Quantum Solutions (59). Source code for GROningen MAchine for Chemical Simulation (GROMACS) 4.5 was downloaded from http://www.GROMACS.org under the GROMACS General Public License (GPL) (45, 46, 60). Automated Topology Builder (ATB) is maintained by the National Computational Infrastructure (NCI) at Australia National University and is freely available at https://atb.uq.edu.au/ (61, 62). A Genetically Optimized Ligand Docking (GOLD) license was obtained from the Cambridge Crystallographic Data Center (63).

## Experimental procedures

### SULT1E1 plasmids

The SULT1E1 expression plasmid consists of an *E. coli* codon-optimized SULT1E1 coding region inserted into the PreScission Protease cleavage site of a triple-tagged pGEX-6P expression vector containing an (N-terminal)-His/GST/MBP tag (43, 64). The cys-insertion mutants used for regiospecific attachment of maleimide-based labels were constructed as follows: cys69 (the only DTNB reactive cysteine) was replaced with ser, and three single-cys mutants were then created by inserting cys into the nonreactive (C69S) scaffold at G16, N150, and N233. The mutations used to test the SULT1E1 structural model (Y239A, F75A, F80A, and F75A/F80A double mutants) were inserted into the WT coding region. All mutations were generated using site-directed PCR mutagenesis (43, 65).

### SULT1E1 purification

*E. coli* (BL21(DE3)) harboring an SULT1E1 pGEX-6P expression plasmid containing a His/GST/MBP triple tag was grown at 37 °C in LB medium (43, 64). At OD_600_ ∼ 0.6, the culture was temperature shifted to 17 °C in an ice/water bath. After the culture reached 17 °C, expression was induced with 0.30 mM IPTG and incubation was continued at 17 °C for 18 h. Cells were pelleted and resuspended in lysis buffer (PMSF (290 μM), pepstatin A (1.5 μM), lysozyme (0.10 mg/ml), EDTA (2.0 mM), KCl (400 mM), K_2_PO_4_ (50 mM), pH 7.5). The suspension was sonicated, centrifuged (10,000*g*, 1.0 h, 4 °C), and the supernatant was collected. MgCl_2_ (5.0 mM) was added to the supernatant to chelate EDTA before passing it through a Chelating Sepharose Fast Flow column charged with Ni^2+^. The column was washed (imidazole (10 mM), KCl (400 mM), and KPO_4_ (50 mM), pH 7.5), and the enzyme was eluted (imidazole (250 mM), KCl (400 mM), and KPO_4_ (50 mM), pH 7.5) and loaded directly onto a Glutathione Sepharose column. The GST column was washed (DTT (2.0 mM), KCl (400 mM), and KPO_4_ (50 mM), pH 7.5), and the tagged enzyme was then eluted (reduced glutathione (10 mM), DTT (2.0 mM), KCl (400 mM), and Tris (100 mM), pH 8.0). The fusion protein was digested overnight at 4 °C using PreScission Protease and passed through a GST column to remove the tag. The protein was ≥95% pure as judged by SDS-PAGE using 2,2,2-trichloroethanol (TCE) treated gels, and its concentration was determined by UV absorbance (Ɛ_280 SULT1E1_ = 61.1 mM^−1^ cm^−1^ (58)). The protein was then concentrated, flash-frozen, and stored at −80 °C.

### Covalent labeling

Labels (N-cyclohexylmale-imide or 3-maleimido-PROXYL) were added in 20-fold excess over reactive cysteine to a solution containing SULT1E1 construct (50 μM active sites), 0.50 mM PAP, and 50 mM KPO_4_ (pH 7.4), 25 °C ± 2 deg. C. PAP was added to enhance enzyme stability. The reactions were monitored by using DTNB to measure unreacted cysteine and were considered complete when >98% of the cysteine had reacted (∼3 h).

### Equilibrium binding studies

The binding of inhibitors to WT and mutant SULT1E1 was monitored *via* ligand-induced changes in the intrinsic fluorescence of the enzyme (λ_ex_= 290 nm, λ_em_= 340 nm) (4). Titrations conditions: OH-PCB (0.10–40 × K_d_) SULT1E1 (15–50 nM, active site), PAP (0 or 300 μM, 100 × K_m_), E2 (0 or 200 nM, 50 × K_d_), KPO_4_ (50 mM), pH 7.5, 25 °C ± 2 deg. C. Titrations were performed in triplicate and the averaged data were least-squares fit to the following quadratic, single-site binding model: I_o_ − ΔI∙(([L]+E_tot_ + K_d_) − [([L]+E_tot_ + K_d_)^2^ − (4∙[L]∙E_tot_)]^1/2^)/2∙E_tot_, where I_o_ and ΔI represent fluorescent intensity at zero and infinite [L].

### Initial-rate studies

#### 1-HP studies

Reactions were initiated by addition of PAPS (0.30 mM, 100 × K_m_) to a solution containing SULT1E1 (20 nM, active sites), 1-HP (4–100 nM, 0.2–5 × K_m_), and KPO_4_ (50 mM), pH 7.5, 25 °C ± 2 deg. C. Reaction progress was monitored *via* the fluorescence change associated with 1-HP sulfonation (λ_ex_ = 325 nm, λ_em_ = 370 nm (51, 52)). Initial rates were measured during conversion to product of ≤5% of the concentration-limiting substrate consumed at the reaction endpoint. Velocities were determined in triplicate. K_m_ and k_cat_ were obtained by (1/v^4^)-weighted least-squares fitting of the averaged data in double-reciprocal space (*i.e.*, 1/v *versus* 1/[S]) (66, 67).

#### OH-PCB1 inhibition studies

Inhibition studies were performed as described above except: 1-HP was fixed (2.0 μM, 100 × K_m_), OH-PCBs were added (0.20–20 × K_i_), and SULT1E1 was 0.20 nM (active sites). K_i_ was determined by least-squares fitting to a noncompetitive inhibition model (66, 67).

#### E2 sulfonation initial-rate assay

SULT1E1-catalyzed conversion of [^3^H]-E2 to [^3^H]-E2-sulfate was quantitated as previously described (68). Reaction conditions: Briefly, WT or F75A/F80 SULT1E1 (0.10 nM, active sites), [^3^H]-E2 (0.40–10 nM, 0.20–5.0 × K_m_, SA = 163 mCi μmol^−1^), KPO_4_ (50 mM), pH 7.5, 25 °C ± 2 deg. C. Reactions were initiated by addition of PAPS (0.30 mM, 100 × K_m_) and quenched after 90 to 180 s with KOH (final concentration 0.10 M). The reaction mixture was diluted tenfold with an E2 (10 μM), KPO_4_ (50 mM), pH 7.5 solution, and brought to pH 7.0 with HCl (6.0 N). The reaction was then mixed (1:1 v/v) with neat chloroform and centrifuged (15,000*g*, 5.0 min). The aqueous layer was removed, chloroform was extracted twice more, and radioactivity in the aqueous layer was then determined by liquid scintillation counting. Reactions were performed in triplicate and K_m_ and k_cat_ were obtained by (1/v^4^)-weighted least-squares fitting of the data in double-reciprocal space (*i.e.*, 1/v *versus* 1/[S]) (67).

### 1H NMR peak assignments

NMR experiments were performed using a Bruker 600 MHz spectrometer equipped with a TCI H/F-cryogenic probe at 298 °K. 1D-proton and 1D-carbon data collection conditions: OH-PCB1 (500 μM), TMS (0.50 mM), D_2_-choloroform (≥99%), 25 °C ± 1 deg. C, spectral windows: 0 to 14 (proton spectra) and 0 to 200 ppm (carbon spectra). Peak assignments were made using ^1^H-^13^C Heteronuclear Single Quantum Coherence (HSQC) (69) and Heteronuclear Multiple Bond Correlation (HMBC) (69).

### Paramagnetic relaxation studies

OH-PCB1 1D-proton spectra were collected under each of the following conditions: SULT1E1 paramagnetic or diamagnetic construct (0, 2.5, 5.0, 10, 25 μM, active sites), OH-PCB1 (50 μM, 833 × K_d_), E2 (4.0 μM + [SULT1E1_active sites_]), PAP (300 μM, 100 × K_d_), KPO_4_ (50 mM), pD 7.4, 25 °C ± 1 deg. C. Peak widths were obtained by fitting to a Lorentzian distribution using NMRdraw (70).

### NMR-distance-restricted molecular dynamics modeling

As described previously (34, 35, 36), a ligand-free model of SULT1E1 was constructed from the SULT1E1⋅PAPS (PDB 1HY3 (71)) structure using SWISS-MODEL. The model was protonated (pH 7.4) and energy minimized using GROMACS. GROMAS57 energy-parameter files were created using Automated Topology Builder (62) for OH-PCB1, PAPS, E2, and a spin-labeled cysteine analogue in which the nitroxyl-moiety was replaced by a hydroxyl group. Spin-labeled cysteine analogues were added as noncanonical amino acids to the GROMAS57 energy field and used to create a triply spin-labeled model in which G16, N150, and N233 are replaced by the analogue. PAPS and E2 were positioned in the active site of the spin-label model using GOLD (72, 73) and the system was equilibrated (298 °K, NaCl (50 mM), pH 7.4) in 100 psec increments using GROMACS. Once equilibrated, OH-PCB1 was randomly positioned in a simulated cube of water (52 × 52 × 52 Å) containing the spin-labeled construct and docked using GROMACS (73). OH-PCB1 docking was constrained using the NMR-determined spin-label/PCB1-proton distances as described in Results and discussion, Refining the structure.

### Transfection protocol

pcDNA 3.1 constructs harboring either the SULT1E1 WT or double mutant (F75A/F80A) coding regions were generated using Gibson Assembly (74). The vectors were linearized using BGIII. Ishikawa cells were grown at 37 °C ± 2 deg. C in growth medium (MEM media containing 10% v/v FBS). At confluence, the cells were washed (3×) with PBS media before coating with an Opti-MEM solution containing a linearized construct (50 ng μg^−1^) and lipofectamine (2.5 units ml^−1^). After 24 h at 37 °C ± 2 deg. C, cells were washed (3×) with PBS before adding growth medium containing neomycin (400 μg ml^−1^) to select transfectants. Selective growth medium was replenished every 48 h until single colonies could be isolated (∼3–6 weeks). Single colonies were transferred using trypsin digest (0.05%) to 12-well plates and grown at 37 °C ± 2 deg. C to confluence for further experimentation and storage.

### Transfectant SULT1E1 levels

Transfectants were grown at 37 °C ± 2 deg. C to 60 to 70% confluency in 12-well plates, washed (3×) with PBS (25 °C), and lysed using RIPA buffer (0.50 ml) (75). Lysate was centrifuged (15,000*g* for 10 min, 25 °C) and the supernatant was collected, assayed, flash-frozen, with liquid nitrogen, and stored at −80 °C. SULT1E1 activity was stable following freeze/thaw. Extract protein concentrations were determined using the Bradford assay (76) and SULT levels were determined by measuring turnover at saturating 1-HP and PAPS. Assay conditions were identical to those described in *Initial-Rate Studies* except that extract (1–3 μg) was added in lieu of pure enzyme.

### Estrogen receptor activation studies

Transfected Ishikawa cells were grown at 37 °C ± 2 deg. C to 60 to 70% confluency in MEM containing 10% v/v FBS. Cells were then transferred to 96-well tissue culture plates and grown at 37 °C ± 2 deg. C to 80% confluency in MEM containing 10% v/v charcoal filtered FBS. The plate was then washed with PBS and grown at 37 °C ± 2 deg. C for 24 h in MEM without FBS. Estradiol (0–3.0 μM) and OH-PCB (0–1.0 μM) solubilized in neat DMSO were added and cells were incubated at 37 °C ± 2 deg. C for 5 days. The final concentrations of DMSO were ≤0.10%. Cells were then washed twice with PBS before adding pNpp (5.0 mM) in PBS (75). Following incubation with pNpp for 3 h at 25 °C ± 2 deg. C, wells were read at 405 nm using a Synergy HT BioTek Plate Reader.

## Data availability

All data and materials are available upon request at tom.leyh@einsteinmed.org. All SULT1E1⋅E2⋅ PAPS⋅OH-PCB1 spin-label directed docking models and the NIH SAVE v5.0 structure validation report are available for download at www.model.archive.org (accession no. ma-xnec7).

## Conflict of interest

The authors declare that they have no conflicts of interest with the contents of this article.
