# Supplementary material for: The molecular basis of OH-PCB estrogen receptor activation
Source: J Biol Chem. 2021 Jan 30;296:100353. doi: 10.1016/j.jbc.2021.100353 (PMC7949139; doi:10.1016/j.jbc.2021.100353)
Supplement: Supplemental Figure S1 [file mmc1.pdf]

The molecular basis of OH-PCB estrogen receptor activation

Ting Wang[§‡], Ian Cook[§] and Thomas S. Leyh[§*]

[§]*Department of Microbiology and Immunology, Albert Einstein College of Medicine, 1300 Morris Park Ave, Bronx, New York 10461-1926.*

[*]*Corresponding Author*

[‡]Current Address: *Drug Metabolism and Pharmacokinetics Department, Boehringer Ingelheim, Danbury, CT*

*Supported by the National Institutes of Health Grants GM121849** and *GM127144**.

Key Words: Polychlorinated biphenyl, PCB, OH-PCB, hydroxylated PCB, sulfotransferase, allosteric, inhibitor, mechanism, SULT1E1, NMR, spin label, structure, molecular dynamics, GROMACS, estrogen receptor, activation

Address: The Department of Microbiology and Immunology
Albert Einstein College of Medicine
1300 Morris Park Ave.
Bronx, New York 10461-1926
Phone: 718-430-2857
Fax: 718-430-8711
E-mail: tom.leyh@einsteinmed.org

Running Title: OH-PCB inhibition of human sulfotransferase 1E1

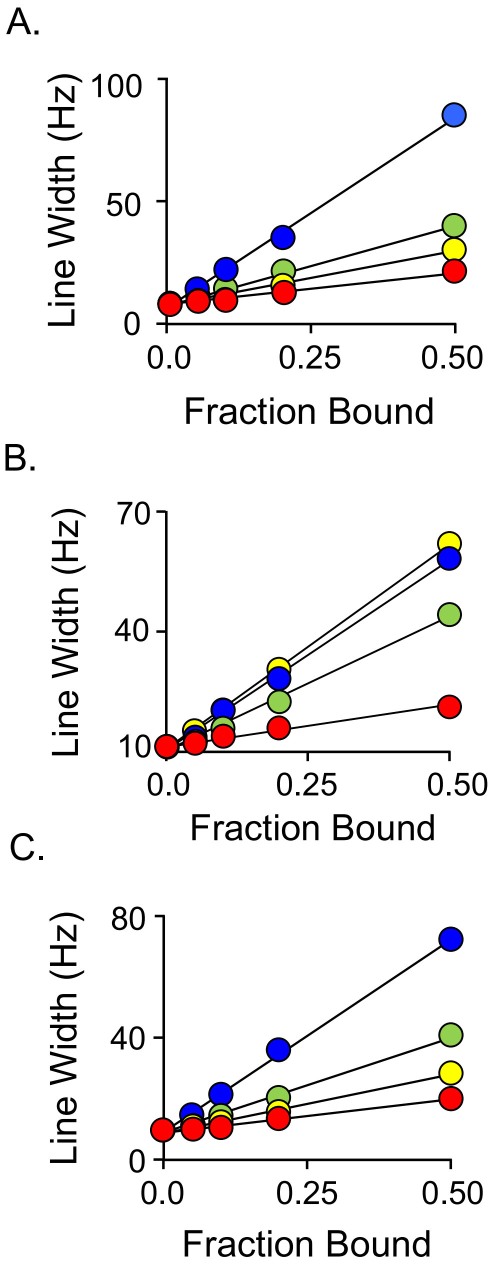

Figure S1. *Line-Width vs Fraction-PCB1-Bound plots.* Panels A, B and C, respectively, present ^{1}H NMR data associated with PCB1 peaks H2 H6, H4' and H3' H5' (see, *Results and Discussion*). Conditions: PCB1 (50 µM), dia- or paramagnetic SULT1E1 construct (0, 2.5, 5.0, 10, 25 µM), E2 (4.0 µM + SULT1E1 monomer concentration), PAP (300 µM, 100 x K_d), KPO_4 (50 mM), pD 7.4, 25 ± 1 °C. Spin-label insertions at positions 16, 233 and 150 are shown in yellow, blue and green, respectively, the control diamagnetic label, seen in red, is inserted at position 233. Each point is the average of three determinations whose standard errors lie within the area of the circle.
